# Cardiothoracic robotic assisted surgery in times of COVID-19

**DOI:** 10.1007/s11701-020-01090-7

**Published:** 2020-05-08

**Authors:** Jef Van den Eynde, Senne De Groote, Robin Van Lerberghe, Raf Van den Eynde, Wouter Oosterlinck

**Affiliations:** 1 Department of Cardiovascular Diseases, Research Unit of Cardiac Surgery, University Hospitals Leuven, Herestraat 49, 3000 Leuven, Belgium; 2 Department of Anesthesiology, University Hospitals Leuven, Leuven, Belgium

**Keywords:** Cardiac surgery, COVID-19, Robotic surgical procedures, SARS-CoV-2, Thoracic surgery

## Abstract

The coronavirus disease 2019 (COVID-19) pandemic poses an immense threat to healthcare systems worldwide. At a time when elective surgeries are being suspended and questions are being raised about how the remaining procedures on COVID-19 positive patients can be performed safely, it is important to consider the potential role of robotic assisted surgery within the current pandemic. Recently, several robotic assisted surgery societies have issued their recommendations. To date, however, no specific recommendations are available for cardiothoracic robotic assisted surgery in COVID-19 positive patients. Here, we discuss the potential risks, benefits, and preventive measures that need to be taken into account when considering robotic assisted surgery for cardiothoracic indications in patients with confirmed COVID-19. It is suggested that robotic assisted surgery might have various advantages such as early recovery after surgery, shorter hospital stay, and reduced loss of blood and fluids as well as smaller incisions. However, electrosurgical and ultrasonic devices, as well as CO2 insufflation should be managed with caution to prevent the risk of aerosolization of viral particles.

The coronavirus disease 2019 (COVID-19) pandemic poses an immense threat to healthcare systems worldwide. Its repercussions are also felt in multiple branches of surgery, with the majority of elective surgeries being suspended to prioritize the use of means, operating rooms, and intensive care beds for COVID-19 positive patients. Questions have been raised as to which surgical procedures can still take place and if they do, how they can be performed safely. In response to this situation, various surgical societies have already issued their recommendations on adequate patient selection and preparation, as well as measures that can be taken to minimize the spread of viral particles. More recently, the European Society of Urology—Robotic Urology Section (ERUS) [1], the American Association of Gynecologic Laparoscopists (AAGL) [2], and the Society of European Robotic Gynaecological Surgery (SERGS) [3] have published their statements on robotic assisted surgery (RAS) in response to the current pandemic. However, to date no specific recommendations are available for cardiothoracic RAS in COVID-19 positive patients.

It has to be noted that most guidelines recommend to suspend all elective procedures, first to create capacity for the care of victims of the pandemic but second to prevent exacerbation of the cytokine storm associated with COVID-19 infection. As all surgical procedures induce a considerable amount of inflammation, this should always be weighed against the benefits of timely intervention. Once a decision has been made after a thorough selection, several measures need to be taken into account during RAS, as summarized in Table 1.

**Table 1:** Measures during cardiothoracic robot assisted surgery (Adapted and modified from Table 3 in Kimmig et al. (2019) J Gynecol Oncol. 31(3):e59)

All surgery during the COVID-19 pandemic should be regarded as high-risk, and, therefore, adequate preventive measures should be taken even in patients who tested negative or who have not been tested for COVID-19
During cardiothoracic robotic assisted surgery, take steps to minimize CO2 release
Close the taps of ports before inserting them to avoid escape of gas during insertion
Attach a CO2 filter (ULPA or similar) or water lock to one of the ports for smoke evacuation. Do not open the tap of any ports unless they are attached to a CO2 filter or being used to deliver the gas
Minimize introduction and removal of instruments through the ports as much as possible. For introduction of material (such as bags, meshes) or specimen retrieval (such as biopsies), deflate the thorax with a suction device before entering or removing the material into or from the thorax or use an air-lock system. Re-insert the port before turning CO2 on again
At the end of the procedure turn CO2 off, deflate the thorax with a suction device and via the port with CO2 filter, before removal of the ports
Avoid the use of ultrasonic sealing and use lowest possible electrocautery power. If possible use electrothermal bipolar vessel sealing
One-lung ventilation should not be used in patients with COVD-19 diseased lungs and PEEP should not be lowered in an attempt to improve surgical visualisation

*COVID-*19 coronavirus disease 2019, *PEEP* positive end-expiratory pressure, *ULPA* Ultra-Low Penetrating Air

As pointed out by ERUS and AAGL, electrosurgical and ultrasonic devices can produce large amounts of smoke. The low-temperature aerosol from ultrasonic scalpels seems to be ineffective in deactivating the molecular components of viruses and other microbial agents [4]. Among others, activated Corynebacterium, papillomavirus, and HIV have been detected in surgical smoke. Gloster et al. [5] reported the transmission of a rare papillomavirus to several healthcare workers after exposure to surgical smoke. To decrease the production of surgical smoke, the power setting of the electrocautery should, therefore, be as low as possible and long dissecting times at the same spot should be avoided.

Furthermore, it has been demonstrated that 10 min of electrocautery creates smoke with higher particle concentrations during laparoscopic surgery when compared to open surgery [6]. A possible explanation might be the relatively low gas mobilisation within a pneumoperitoneum. Moreover, the increased intracavitary pressures associated with pneumoperitoneum might represent an additional risk for aerosolization of viral particles with potential exposure of the operating staff. Because similar principles are applied for CO2 insufflation of the thorax, it is important to also be aware of this theoretical risk of viral spread in cardiothoracic RAS. Viral load might potentially even be higher in operating fields close to the lungs, especially because COVID-19 lesions are mostly seen at the basal and peripheral parts of the lungs [7]. Insufflation pressures should, therefore, be reduced to the lowest level possible without compromising the surgical field exposure; research has demonstrated that robotic vision remains stable and optimal up to 5 mmHg [8]. Care should also be taken to prevent sudden release of trocar valves or non-air-tight change of instruments. Additionally, it is advised that an integrated flow system is used with continuous smoke evacuation through an Ultra-Low Penetrating Air (ULPA) filter or water lock to avoid escape of gases and particles [3]. If an incision needs to be made after the robotic assisted part of the procedure, such as is the case in robotic assisted minimally invasive coronary artery bypass grafting, CO2 insufflation should be stopped and intrathoracic pressures should have decreased sufficiently before the incision can be made.

Importantly, cardiothoracic RAS may require one-lung ventilation to create more space within the thoracic cavity, hence providing adequate visualization during robotic surgery. However, the use of one-lung ventilation may constitute several dangers for both the patient and healthcare workers. First, airway instrumentation, including in- and extubation maneuvers as well as bronchoscopy, for the placement of a double lumen tube (DLT) or endobronchial blocker represents additional risk of aerosolization of viral particles [9]. Second, one-lung ventilation may be associated with intolerable hypoxemia or hypercapnia in COVID-19-injured lungs [10]. Third, one-lung ventilation is associated with additional lung injury, even when protective one-lung ventilation strategies are used [11]. To date, no clinical studies have addressed these dangers, but it may be advisable to avoid one-lung ventilation in COVID-19 diseased lungs if possible. Lowering the positive end-expiratory pressure (PEEP) in patients with acute respiratory distress syndrome (ARDS) in an attempt to create more space and better surgical visualization is also not a good option, as this will result in hypoxemia [12].

Provided that the above discussed risks are taken into account and met with these preventive measures, cardiothoracic RAS might on the other hand have various benefits to offer during the current COVID-19 pandemic when compared to conventional open surgery. First, RAS allows for earlier mobilization of the patient and a shorter hospital stay, resulting in lower chances of patients contracting complications such as pneumonia or spreading COVID-19 at the ward. In addition, more hospital beds will be preserved to meet the increased need of capacity for the treatment of non-surgical COVID-19 positive patients. Furthermore, blood and fluid loss are considerably lower after RAS than after open surgery and incisions are smaller, thus implying less routes through which the virus can spread to healthcare professionals, other patients, and the hospital environment.

Offering a type of surgery which includes less direct tissue contact, which is performed in a closed system, and which is characterized by early postoperative recovery, RAS might be an option with regard to patients requiring cardiothoracic surgery in times of COVID-19, although only after thorough selection based on patient characteristics and severity of the condition that requires treatment. Caution is advised with the use of electrosurgical and ultrasonic devices, CO2 insufflation, and the use of trocar valves, all of which carry a potential risk of aerosolization into the operating theatre. Adequate measures including filtration systems should, therefore, at any time be respected when performing RAS in COVD-19 positive patients. Furthermore, one-lung ventilation should not be used in COVID-19 diseased lungs. Finally, general measures recommended by surgical societies such as personal protective equipment, optimal patient selection, and limitation of operating room staff evidently remain applicable in RAS and should be adhered to strictly.
